# An Evolutionary Approach to Passive Learning in Optimal Control Problems

**DOI:** 10.1007/s10614-019-09961-4

**Published:** 2020-01-02

**Authors:** D. Blueschke, I. Savin, V. Blueschke-Nikolaeva

**Affiliations:** 1 University of Klagenfurt, Klagenfurt, Austria; 2 Institute of Environmental Science and Technology, Universitat Autónoma de Barcelona, Cerdanyola del Vallès, Spain; 3 Graduate School of Economics and Management, Ural Federal University, Yekaterinburg, Russian Federation

**Keywords:** Optimal control, Stochastic problems, Differential Evolution, Passive learning

## Abstract

We consider the optimal control problem of a small nonlinear econometric model under parameter uncertainty and passive learning (open-loop feedback). Traditionally, this type of problems has been approached by applying linear-quadratic optimization algorithms. However, the literature demonstrated that those methods are very sensitive to the choice of random seeds frequently producing very large objective function values (outliers). Furthermore, to apply those established methods, the original nonlinear problem must be linearized first, which runs the risk of solving already a different problem. Following Savin and Blueschke (Comput Econ 48(2):317–338, 2016) in explicitly addressing parameter uncertainty with a large Monte Carlo experiment of possible parameter realizations and optimizing it with the Differential Evolution algorithm, we extend this approach to the case of passive learning. Our approach provides more robust results demonstrating greater benefit from learning, while at the same time does not require to modify the original nonlinear problem at hand. This result opens new avenues for application of heuristic optimization methods to learning strategies in optimal control research.

## Introduction

Mathematical models are pervasive in economics. They are often given in a form of a dynamic system of equations describing how economy evolves over time. Since it is widely accepted that the nonlinear framework allows to derive more precise picture of reality compared to the linear one, we consider a system of nonlinear equations describing an economy. Having such a mathematical system, one is tempted to use it in order to optimize the state of the world or rather to guide it into a desired direction. This is a very simplified description of the optimal control framework. One important topic in this research field is the inclusion of learning strategies. We follow this line of research and introduce in this study a new way for including a passive learning strategy into an optimal control problem. The term “learning” used here implies that the policy-makers (or any other user of optimal control problems) are willing to observe and to learn from the current state of the world. As a result, the information about the system is updated systematically. Thus, in addition to the optimal control process an updating procedure is included in this methodology.

The basic work on introducing learning in the stochastic optimal control field can be found in the studies by Tse and Bar-Shalom (1973) and Kendrick (1981). There are two main concepts of learning, namely passive learning (or open-loop feedback) and active learning (see Kendrick and Amman (2006) for a common classification). Some more recent work on the latter topic can be found in Beck and Wieland (2002), Amman et al. (2018) and Amman and Tucci (2018). In this study we concentrate on the passive learning approach. However, an extension of the proposed evolutionary method to include the active learning is an obvious path for the future research.

By considering the passive learning strategy we follow the approach introduced by Kendrick (1981).^1^ However, this framework was restricted to linear models only. Later, it was extended to nonlinear problems in Blueschke-Nikolaeva et al. (2012) and resulted in the algorithm OPTCON2. In this study, OPTCON2 is used as a “traditional” baseline solution to check the quality of the new proposed methodology. The feature of the OPTCON2 algorithm is a simultaneous consideration of nonlinear systems and passive learning strategy. The passive learning occurs using the Kalman filter update procedure. The nonlinear issue is solved by an iterative approximation procedure using the linear-quadratic (LQ) framework. Some common limitations for the linear-quadratic (LQ) framework include an symmetric objective function and very limited ability for additional restrictions like inequality constraints. Many of this issues were already solved by replacing the linear-quadratic (LQ) framework by the Differential Evolution method. In particular, the restriction of the symmetric objective function are addressed in Blueschke et al. (2013b), while inequality constraints—in Savin et al. (2018). In addition, Blueschke and Savin (2017) compare alternative forms of objective function specification, which may be more suitable if—next to achieving policy targets—one is also willing to minimize volatility of the economic system over time.

A more general limitation for the linear-quadratic (LQ) framework applied to nonlinear problems is the need to linearize them first. This is particularly true for stochastic problems, where some simplification assumptions on transferring the stochastic information from the nonlinear to the linear model have to be made. As it has been argued by Savin and Blueschke (2016), this causes a loss of information, where the linearized objective function of the model at hand may not necessarily correspond to the actual problem anymore. In fact, the need to (over)simplify the actual problem before being able to solve it is quite common in the literature (see Gilli and Schumann (2014) for an overview). An alternative to problem simplification is to modify the optimization algorithm, seeking not necessarily a theory-based derivative-depending optimum but only its approximation, i.e. a solution “good enough” to satisfy the requirements to the problem [e.g., reach a loss value below a given threshold, Gilli and Schumann (2011)]. By sacrificing optimality, however, one gains in terms of flexibility with respect to additional assumptions imposed on the problem (as heuristics do not have any) and sometimes speed (as in certain cases^2^ an exact solution could be prohibitively expansive). A family of computational algorithms that do not require to modify the original (nonlinear) problem but iteratively approximate its optimum until some stopping criterion is not met has been framed in the literature “heuristic” methods. And Differential Evolution (DE, henceforth) is one of those methods designed for complex continuous problems. The first time DE has been applied to optimal control problems was by Blueschke et al. (2013b) demonstrating its flexibility to introducing asymmetric penalties for deterministic optimal control problems. Later, DE has been extended to stochastic problems in Savin and Blueschke (2016). The present study offers a first application of DE to optimal control with learning.

The main motivation of this study is to combine a promising field of evolutionary-based optimization techniques such as DE with a sophisticated perception of the reality, namely the ability of decision-makers to learn during the optimal control process. We are well aware that the computational costs of such an approach are expected to be very high. However, it would allow for a more flexible framework to be used.

The rest of the paper is structured as follows. In Sect. 2 the theoretical background is briefly described. It includes a description of an underlying optimal control problem and a traditional way to solve them with passive learning strategy. Section 3 discusses the evolutionary (DE-based) approach and introduces its extension to passive learning. An application example is presented in Sect. 4. Section 5 concludes.

## Theoretical Background

We consider a nonlinear dynamic system given as difference equations of the following form:

1 $$\begin{aligned} x_t=f(x_{t-1},x_t, u_t, \theta , z_t)+\varepsilon _t, t=1, \ldots , T, \end{aligned}$$

where $x_t$ is an *n*-dimensional vector of state variables that describes the state of the economic system at any point in time *t*. $u_t$ is an *m*-dimensional vector of control variables that are exogenous for the system. However, they are directly controlled by the decision-maker and are used to influence the state of the world. $z_t$ denotes a vector of non-controlled exogenous variables, $\theta$ is a *p*-dimensional vector of parameters, which values are assumed to be constant but unknown to the decision-maker (parameter uncertainty) and $\varepsilon _t$ is an *n*-dimensional vector of additive disturbances (system error). $\theta$ and $\varepsilon _t$ are assumed to be independent random vectors with expectations $\hat{\theta }$ and $O_n$ and covariance matrices $\varSigma ^{\theta \theta }$ and $\varSigma ^{\varepsilon \varepsilon }$, respectively. *T* denotes the terminal time period of the finite planning horizon.

The decision-maker uses the variable $u_t$ to achieve, or rather to approximate, a desired state of the world. All her targets are summarized in the objective function as follows:

2 $$\begin{aligned} J=E \left[ \sum ^T_{t=1}L_t(x_t,u_t) \right] , \end{aligned}$$

with

3 $$\begin{aligned} L_t(x_t,u_t)=\frac{1}{2}\left( \begin{array}{c} x_t-\tilde{x}_t\\ u_t-\tilde{u}_t\\ \end{array} \right) 'W_t\left( \begin{array}{c} x_t-\tilde{x}_t\\ u_t-\tilde{u}_t\\ \end{array} \right) . \end{aligned}$$

$\tilde{x}_t\in R^n$ and $\tilde{u}_t\in R^m$ are given desired (target) levels of the state and control variables, respectively. $W_t$ is an $((n+m)\times (n+m))$ matrix specifying the relative weights of the state and control variables in the objective function.

Optimal control task can be seen as a minimization problem, which means a path of control variables $u_t$ must be calculated such as to minimize the objective function (2). However, this optimization task is restricted by the dynamics of the system (1). In this study an optimization approach with passive learning strategy is considered. An inclusion of the passive learning strategy (also known as open-loop feedback, OLF) means that the information about the underlying “true” system as given by parameter $\theta$ is updated in each time period as a result of the observed “true” state of the world at the “end of the day”.

### Passive Learning (OLF)

The term “passive learning” used in this study means observing the current state of the world and using the new information to improve the knowledge of the system. The idea of passive learning (OLF) can be explained as follows. The policy-maker does not know the “true” parameter vector $\widehat{\theta }$ of the model and works with a set of its (imperfect) empirical estimates $\theta ^k$ (see Sect. 3.2). Every period, the policy-maker derives the optimal path of the control variables $u^{*}$ as a function of the parameter $\theta ^k$, and not $\widehat{\theta }$. And the state variables are calculated correspondingly $x^{*}=f(u^{*}, \theta ^k, \ldots )$. At the end of each time period, the decision-maker observes what has happened in reality, i.e. the outcome based on the “true” model ($x^{a*}=f(u^{*}, \widehat{\theta }, \ldots )$) and not the estimates of the policy-maker. The policy-maker then applies this information (or rather the mismatch between predicted and actual state of the system due to the difference between $\theta ^k$ and $\widehat{\theta }$) to re-estimate the model and to update the system, $\theta ^k_{new}=\mathcal {F}(x^{a*}, x^{*},\theta ^k)$. By $\mathcal {F}$ we denote a procedure which is used for the update of parameters. There are different alternatives for the update procedure. In the OPTCON2 algorithm the Kalman filter is used for this purpose.

### OPTCON2

The idea of the paper is to introduce a new evolutionary-based approach for obtaining OLF solutions of the optimal control problem (see Eqs. 1–3). To test the performance of the new approach (introduced in Sect. 3) we use the OPTCON2 algorithm as a baseline. The advantage of OPTCON2 is that it can be applied to both, open-loop (OL) and open-loop feedback (OLF) type of problems. For the latter, an additional updating procedure based on the Kalman filter is included at the end of each time period of the optimization. Furthermore, as mentioned above, it can work with the nonlinear systems. The problem with the nonlinear system is tackled iteratively, starting with a tentative path of state and control variables. The solution is sought from one time path to another until the algorithm converges or the maximal number of iterations is reached. During this search the system is linearized around the previous iteration’s result as a tentative path and the problem is solved for the resulting time-varying linearized system. The approximately optimal solution of the problem for the linearized system is found under the above-mentioned simplifying assumptions about the information pattern; then this solution is used as the tentative path for the next iteration, starting off the procedure all over again. Every iteration, i.e. every solution of the problem for the linearized system, uses the Bellman’s principle of optimality in order to minimize objective function under system conditions. See Blueschke-Nikolaeva et al. (2012) for more details.

## Differential Evolution with OLF

### Differential Evolution

As mentioned earlier, heuristic algorithms are called this way because of their simplified nature not necessarily finding a true optimum solution, but their approximation satisfying certain stopping criteria. These optimization methods are based on evolutionary, nature-inspired, processes employing the principles of randomness, recombination of available solutions, and preserving the fittest out of them. To assure good quality of approximation, heuristics normally have to run a large number of iterations with several restarts. Hence, their widespread use has become possible only in the last few decades thanks to the recent advances in computing technology. These evolutionary methods are designed to address complex optimization problems eligible for various constraints both, in discrete and continuous search spaces. An excellent overview of these techniques is provided by Gilli and Winker (2009).

Differential Evolution (Storn and Price 1997) is a population based optimization technique designed for continuous objective functions with multiple local minima. Based on the idea of genetic evolution, DE uses cooperation and competition of individual solutions, and has only few parameters to tune. In Blueschke et al. (2013b, pp. 824–825) we describe how DE deals with optimal control problem for a deterministic scenario (single parameter set $\theta$). In the following, a short summary of it is provided. In particular, starting with an initial population of random solutions, $P_{j,t,i}^{(1)}$ (line 2 in Algorithm 1), DE updates this population by linear combination (line 7) and crossover (line 10) of four different solution vectors into one, and selects the fittest solutions among the original and the updated population. This continues until some stopping criterion is met. Each candidate solution contains all control variables for all time periods. Thus, each candidate represents an alternative complete solution path for the whole optimum control problem, and is given as an ($m\times T$)-matrix, where *m* is the number of controls and *T*—the size of the planning horizon. Each candidate solution is also described by the time paths of corresponding state variables, which results from the dynamic system *f* in (1), parameter set $\theta$ and the selected controls. For each set of control variables and for each parameter set $\theta$ there is a unique set of state variables, which are not directly included in a candidate solution but contribute to the objective function in (2, 3).
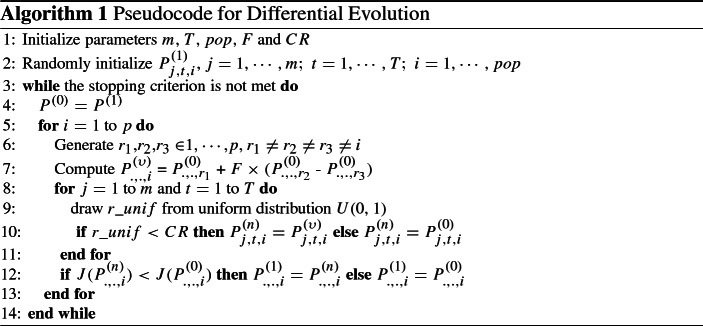


In the present work we apply an extended framework for stochastic problems as described in Savin and Blueschke (2016). In this case DE has to deal in a stochastic set-up not with a single $\theta$, but a set of different $\theta$s representing alternative possible realizations of parameters. We start with an initial DE population $P_{j,t,i}$ (those containing alternative sets of controls *u* to solve the system in Eqs. 1–3). However, we do not minimize a single objective value (for a single $\theta$ draw) for each member of the DE population, but an expected stochastic objective value. This means that we create for each member of the DE population a large number ($\varLambda$) of Monte Carlo realizations of $\theta$ and run for each of them the Algorithm 1 all over again. In the end we calculate the expected objective function value for an individual member of the DE population as a median value across all this possible (stochastic) realizations of $\theta$s. We employ median instead of mean to get a measure robust to outliers. Let us think of a very simple example of an initial population with just two members $u_1$ and $u_2$ ($pop=2$). For each member we create a Monte Carlo set consisting of three different realizations of $\theta$ ($\varLambda =3$). We create for $u_1$ three realizations of $\theta$, run three times the Algorithm 1 and calculate the stochastic solution based on the least median value. Then the same is applied for the $u_2$. The resulting objective functions are used to compare the performance of $u_1$ and $u_2$.

### DE with OLF

The novelty of the present paper is to solve the optimal control problem (1)–(3) using the OLF strategy by means of Differential Evolution. As already discussed in Sect. 2.1, the policy-maker does not know the true parameter of the model $\widehat{\theta }$ and derives the optimal path of the control variables and the corresponding state of the system as a function of its (imperfect) empirical estimate $\theta ^k$. Using the given information the policy maker calculates an optimal stochastic solution according to the Algorithm 1 and the framework described in the last paragraph of Sect. 3.1. The obtained optimal result for the time period $t=1$ and $u^*_1$ is applied. At the end of each time period, she observes the realized (true) values of the state variables and applies this information to re-estimate the model and to update the system. After that the updated $\theta ^k$ is used to find a stochastic solution for the remaining time periods in the planning horizon $t=2, \ldots , T$. Thus an optimal control problem with the OLF strategy calculated by the DE algorithm consists of an iterative procedure as described in Algorithm 2.
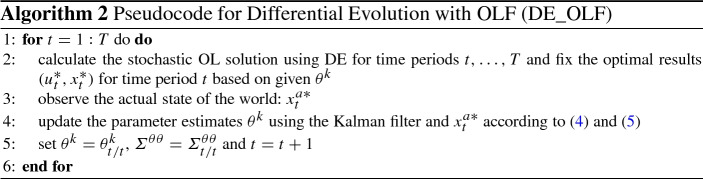


In order to perform the update, the Kalman filter is used, which consists of two steps, prediction and correction:

1. Prediction
  4 $$\begin{aligned} \begin{aligned} \hat{x}_{t/t-1}&=f\left( x^{a*}_{t-1}, \hat{x}_{t/t-1}, u_t^{*},\theta ^k_{t-1/t-1}, z_t\right) =x^*_t,\\ \theta ^k_{t/t-1}&=\theta ^k_{t-1/t-1}, \\ \varSigma ^{xx}_{t/t-1}&=\partial _x f_{\theta t-1}\varSigma ^{\theta \theta }_{t-1/t-1}(\partial _x f_{\theta t-1})'+\varSigma ^{\varepsilon \varepsilon }_t,\\ \varSigma ^{x \theta }_{t/t-1}&=\left( \varSigma ^{\theta x}_{t/t-1}\right) '=\partial _x f_{\theta t-1}\varSigma ^{\theta \theta }_{t-1/t-1},\\ \varSigma ^{\theta \theta }_{t/t-1}&=\varSigma ^{\theta \theta }_{t-1/t-1}. \end{aligned} \end{aligned}$$
  $\partial _x f$ denotes the partial derivative of the function *f*.
2. Correction
  5 $$\begin{aligned} \begin{aligned}&\varSigma ^{\theta \theta }_{t/t}=\varSigma ^{\theta \theta }_{t/t-1}-\varSigma ^{\theta x}_{t/t-1}\left( \varSigma ^{xx}_{t/t-1}\right) ^{-1}\varSigma ^{x\theta }_{t/t-1}\\&\hbox {and} \\&\theta ^k_{t/t}=\theta ^k_{t/t-1}+\varSigma ^{\theta x}_{t/t-1}\left( \varSigma ^{xx}_{t/t-1}\right) ^{-1}[x^{a*}_{t}-x^*_t] \\&\hat{x}_{t/t}=x_t^{a*}. \end{aligned} \end{aligned}$$

Thus, the update procedure allows to obtain the new values $\theta ^k_{t/t}$ and $\varSigma ^{\theta \theta }_{t/t}$. These values are then used in the next time period of the optimal control problem.

Algorithm 2 describes the steps how to obtain an OLF optimal control solution using the Differential Evolution technique (we refer to it hereafter as DE_OLF). As we suggest a novel methodology for optimal control with passive learning, it is important to test its performance compared to an established benchmark (OPTCON2). However, for the update procedure one has to observe the current state of the system which is crucial for the learning procedure. For this reason we use the Monte-Carlo simulations. In this way, some “quasi-real” values can be created and used for comparison between DE_OLF and OLF.^3^ In addition, we can also compare the performance of DE_OLF vs DE_OL (stochastic solution without learning as presented in Sect. 3.1).

For the purpose of comparing OPTCON2 with passive-learning (OLF) and DE with passive learning (DE_OLF), a large number (*K*) of random time paths for the additive ($\varepsilon _t^k$) and multiplicative errors ($\mu ^k$) are generated. $\mu ^k$ are used to calculate the (imperfect) empirical estimates $\theta ^k$ with which the decision-maker works as she does not know the true parameter $\hat{\theta }$. Additive disturbances ($\varepsilon _t^k$) are added to the calculated outcome of the system representing some unpredictable shocks. All together this allows to test how new information can affect performance of the system in “quasi-real” observations. For better understanding, a brief scheme is sketched in Algorithm 3.
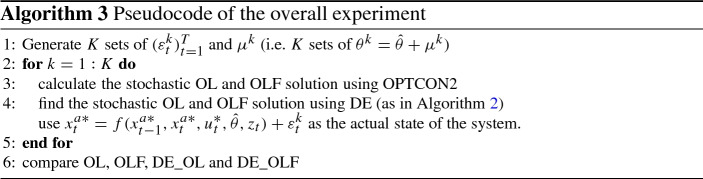


### Tuning and Implementation

Since in this study we will run the model for very different $\theta$ realizations, instead of tuning DE parameters (crossover probability *CR* and differential weight *F*) for any particular subset of them, we take their default values $CR=0.8$ and $F=0.8$ (Storn and Price 1997). Similarly, the size of the population of DE candidate solutions $P_{j,t,i}^{(1)}$ is taken ten times bigger than the number of uncertain parameters, while DE runs for $g^{max}=1000$ generations if an alternative stopping criterion is not met.^4^ Those alternatives stopping criteria include either that 50% of solutions in the DE population reach a deviation of 0.0001% from the best solution available so far, or that for 100 generations at least half of solutions in the population does not improve anymore. These additional criteria, thus, stop DE once its convergence is observed, and are meant to optimize the computational cost of the problem (Blueschke et al. 2013b). Given the stochastic nature of DE, we also restart the DE algorithm three times (i.e. construct anew a different population of candidate random solutions $P_{j,t,i}^{(1)}$ for the same random disturbance and the same population of Monte Carlo realizations of $\theta$) to ensure that it converges, if not to global optimum, to a good approximation of it.

In order to specify the initial set of random parameters of the Monte Carlo experiment, the known information about the variance is used ($\varSigma ^{\theta \theta }$ and $\varSigma ^{\varepsilon \varepsilon }$). It should be mentioned that this information is taken from the nonlinear system directly and no linearization steps are applied. By generating the initial population of control variables of each individual DE optimization, there is always a trade-off to be considered. In particular, one can choose a rather large volatility and explore more possible solutions or, in contrast, restrict it to speed up convergence. We prefer the former option and use all known information such as $u_{0}, \tilde{u}, u_{OPTCON2}$ to allow for a large search space.

Both methods, OPTCON2 and DE, are implemented in MATLAB to simplify their comparison. In this particular study we need to run DE over two Monte Carlo experiments. The first experiment consists of *K* draws of random disturbances (as explained in Algorithm 3) to obtain “quasi-real” observations and to compare DE_OLF, DE_OL, OLF and OL solutions. The second Monte Carlo experiment is applied every time when an individual (stochastic) DE solution needs to be calculated. Thus, the second Monte Carlo experiment consists of $\varLambda$ random draws of possible realizations of parameter $\theta$.

In line with Savin and Blueschke (2016), we keep $\varLambda =1000$, which greatly increases the cost of computation for DE. For that reason, we parallelized computations over alternative $\varLambda$ draws since those can be performed independently. Furthermore, we also parallelize computations for alternative *K* draws of random disturbances by performing our experiments in separate sessions on the high performance computing (HPC) cluster BwUniCluster https://www.bwhpc-c5.de/wiki/index.php/Category:BwUniCluster. However, remembering that due to passive learning we have not *T* but ${ T+1 \atopwithdelims ()2}$ optimization periods,^5^ that have to be performed for up to $g^{max}$ generations, the cpu cost of solving stochastic OLF problem with DE is still very high. In particular, even using a computer with 16 cores running in parallel on a single draw of random disturbances *k*, one DE restart for the ATOPT model described below takes approximately twelve hours to converge.

## Application

### ATOPT Model

We consider a very simple and novel nonlinear dynamic model of the Austrian economy targeting on the output—public debt trade-off (ATOPT). The aim of our study is not the economic interpretation of the solution, but analysis of the DE performance as a solution technique for an optimal control problem with passive learning. Thus, we try to keep the model as simple as possible but allowing for a complete analysis of the topic of interest. The model consists of three endogenous variables: output growth—$y_t$, public debt— $d_t$, and interest rate—$r_t$. These are defined in the following way:

6 $$\begin{aligned} y_t= & {} a_1\cdot y^{world}_t - \theta _1 \cdot g_t + \varepsilon _{1,t} \end{aligned}$$

7 $$\begin{aligned} d_t= & {} (1 + r_t) \cdot d_{t-1} - g_t + \varepsilon _{2,t} \end{aligned}$$

8 $$\begin{aligned} r_t= & {} r_{t-1} + \theta _2\cdot (y_t-\bar{y}_t) + a_2\cdot (d_t - \bar{d}_t)^3 + \varepsilon _{3,t} \end{aligned}$$

As a small open economy, Austria’s growth rate of GDP heavily depends on the economic situation in the world via exports. In order to hold the model system close to reality, this is captured using the correlation coefficient ($a_1$) between Austria’s and world’s GDP growth (between 1996 and 2017) and is equal to 0.7266. In addition, the government is able to use its fiscal policy instrument ($g_t$), which is primary fiscal surplus (or deficit if negative). An expansionary use of the fiscal policy instrument has a positive effect on the output growth, but increases the public debt ($d_t$). An additional driver of the public debt is the interest rate ($r_t$) which has to be paid for the bond holders. The interest rate itself is influenced by both, an excess output growth ($y_t-\bar{y}_t$) and by a risk premium for an excess public debt level ($d_t - \bar{d}_t$). The latter is given in a cubic form to penalize deviations from the acceptable debt level ($\bar{d}$). For the Austrian economy, the acceptable debt level is assumed to be given by the Maastricht criterion of 60% of GDP. The cubic function penalizes less (as compared to the linear one) small deviations around the target level, but much stronger larger deviations from it. This allows for the possibility of financial markets teaching the government fiscal discipline, but it takes into account that there is no exact acceptable level of public debt. As a threshold for the normal output growth (which defines the current output growth to be excessive) a value slightly above the historical average (1996–2017) of 1.84 is assumed here, namely 2% annual growth ($\bar{y} = 0.02$).

The fiscal multiplier parameter ($\theta _1$) is one of the two stochastic parameters in the model. The choice of the fiscal multipliers is a tricky issue as there are many factors which influence this parameter [see, e.g., the discussion in Ilzetzki et al. (2013) and Nakamura and Steinsson (2014)]. We assume the value of $\theta _1$ to be 1.2 which is significantly below the 1.6 value used by Romer and Bernstein (2009), but above the rather low values which are frequently derived in the new classical economic theory. However, to account for the fact that an exact value is highly questionable and different studies derive rather large differences, we assume the variance of the fiscal multiplier to be relatively high ($\varSigma ^{\theta _1}=1$). The second stochastic parameter is the link between output growth and its impact on the interest rate ($\theta _2$). We assume it to be 0.1 with the variance $\varSigma ^{\theta _2}=0.2$.

Equations (6–8) give a very simplified description of the Austrian economy with an output growth—public debt trade-off. Using its instrument, namely fiscal policy *g*, the government aims at maintaining a high GDP growth of 3% ($\tilde{y}=0.03$) and a steady decrease of the public debt from 78.4% of GDP in 2017 to 60% of GDP at the end of the planing horizon, namely in 2022 ($T=5$). At the same time, the government prefers to have a balanced budget ($\tilde{g}=0$). The former two represent the two state variables of the ATOPT model, while the latter variable is the control. Thus, the task is to find an optimal path of the control variable, in order to minimize the difference between the outcome of the system and the given targets. This optimal control problem should be solved using the passive learning strategy.

### Results of Comparison Between OPTCON2 and DE

**Fig. 1:**
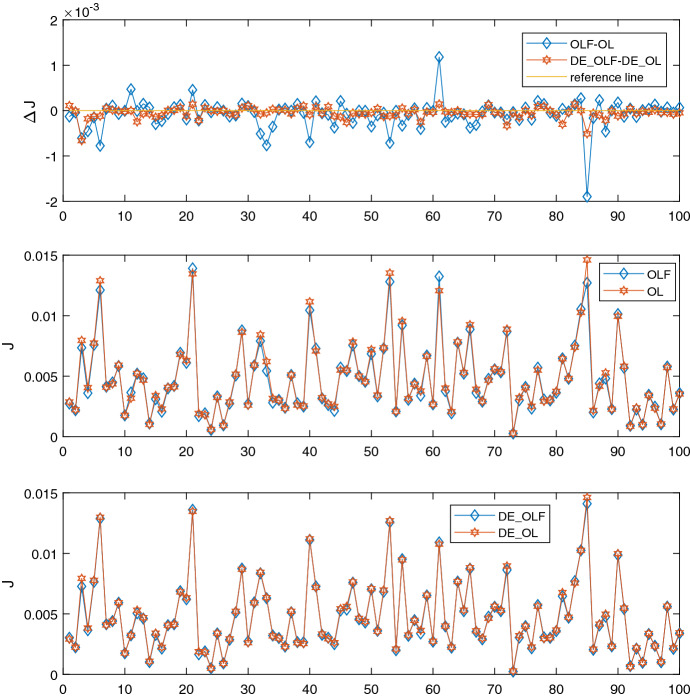
Differences in objective function between OLF and OL solutions (upper panel), OLF vs. OL value of objective function using OPTCON2 (middle panel) and DE (bottom panel)

The main motivation of this study was to show the ability of an evolutionary-based method such as DE to include a more sophisticated (passive) learning strategy. However, the question which should be raised here is about the reason why to use this new method which is more time consuming, if we just replicate the results of the OPTCON2 algorithm? To this end, some known advantages of the DE method were discussed in Sect. 1. In particular, using DE one can extend the standard optimal control framework with an asymmetric or a non-quadratic objective function, and to include additional (inequality) constraints. In addition, we suggest that the new method is more robust to the outliers problem known in the optimal control experiments with learning strategies (see Tucci et al. (2010) and Blueschke et al. (2013a) for a detailed discussion of the topic). To demonstrate this, we use the ATOPT model and run a Monte Carlo experiment consisting of *K* draws of random disturbances (as explained in Algorithm 3) to compare DE_OLF, DE_OL, OLF and OL solutions. Figure 1 shows the graphical results for such an experiment with $K=100$. In particular, we sample random disturbances, *K*, to be equally distant for the intervals $\theta _1 \in [-1,3]$ and $\theta _2 \in [-0.2,0.6]$

One can see from Fig. 1 very similar patterns from comparison between DE_OLF vs. DE_OL and OLF vs. OL results. It should be mentioned, that the Monte Carlo experiment is constructed in such a way, that all solution strategies work with the same set of random disturbances which also allows a direct comparison between DE and OPTCON2 solution. If we look at the absolute numbers, we see that for OPTCON2 OLF gives a better (lower) objective value in 64% of the results. For DE this number is slightly higher: DE_OLF performs better in 68% of the runs as compared to DE_OL.^6^ Furthermore, if we sum up for the upper plot in Fig. 1 all the positive deviations $J_{DE\_OLF} - J_{DE\_OL}$ and $J_{OLF} - J_{OL}$, for OPTCON2 it will amount to 0.0056 while it is three times smaller for DE (0.0018). That means, that using DE passive learning has much lower chance to produce worse results than without learning. For negative deviations DE also has overall smaller value: $-0.007$ vs. $-0.014$ for OPTCON2 implying that also the improvements in terms of objective function values under DE are smaller. These results show that while obtaining similar results by using DE with passive learning as in the case with the derivative-based OPTCON2 algorithm, we avoid large discrepancies between our objective function values with and without learning (OLF vs. OL).

**Fig. 2:**
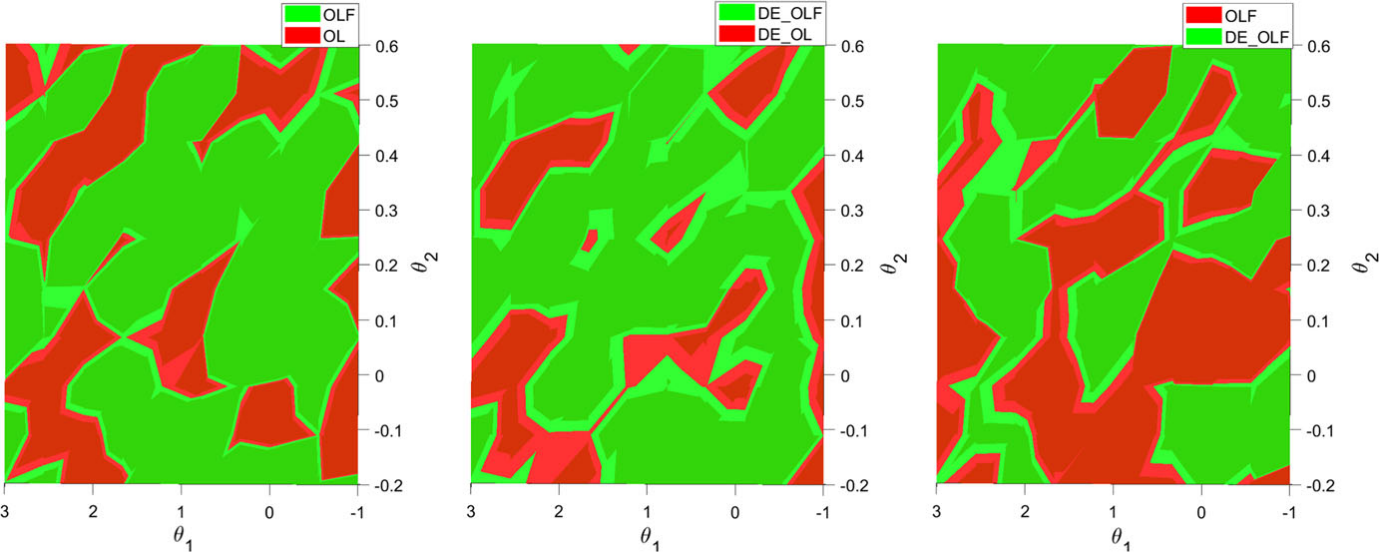
Differences in objective function between OLF and OL solutions using OPTCON2 (left panel), DE_OLF vs. DE_OL (middle panel) and OLF vs. DE_OLF (right panel) for different values of $\theta _1$ and $\theta _2$. Note: the color indicates which of the two options has a lower (superior) objective function value

To see for which combination of $\theta _1$ and $\theta _2$ DE provides better results than OPTCON2, consider Fig. 2. Clearly, for larger values of $\theta _2$ DE_OLF results in smaller objective function values than OLF.

**Fig. 3:**
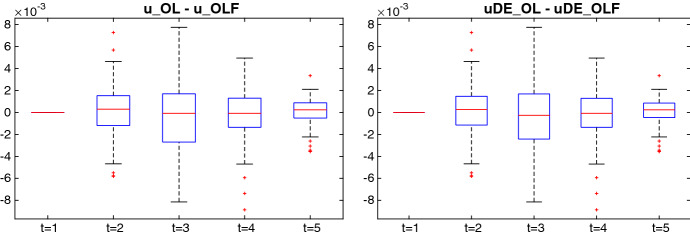
Differences in controls between OLF and OL solutions using OPTCON2 (left panel) and between DE_OLF and DE_OL solutions (right panel)

Finally, to get some insight on how the use of the solution algorithm affects the optimal choice of the controls we plot in Fig. 3 the resulting differences in controls for the ATOPT model. We consider the differences between controls calculated by OL and OLF (left panel) and DE_OL and DE_OLF (right panel) using box-plots for each of the five periods. Each box-plot represents the results across all random disturbances *K* tested ($K = 100$). Interestingly, most of the differences in controls due to open-loop feedback concentrate around periods 2–4 and not in period 5, which one could expect because of iterative nature of solution with deviations accumulating over time. A possible explanation is that this is due to the curse of the last period in a finite time horizon problem. Fortunately, the results between OLF and OL both for OPTCON2 and DE are very similar. Given that the control variable is fiscal surplus (fiscal deficit if it is negative), under open-loop feedback we see a slightly more active fiscal policy in periods 2 and 5 and a more restrictive policy in periods 3 and 4.

## Conclusion

In this study we propose an evolutionary-based method for solving optimal control problems with passive learning. We extend the work in Savin and Blueschke (2016) which introduced a possibility for solving stochastic optimal control problems using the Differential Evolution (DE) method. Using a small econometric model of the Austrian economy and a series of large Monte Carlo experiments we show that the new method produces similar results as a baseline traditional method. The main advantage of using DE is its flexibility allowing to take many restrictions into account. In addition, in case of the open-loop feedback scenario, DE proves to be more robust towards the outlier problem known in the optimal control field with learning.

For the readers, a legitimate question arises what explains the differences in performance between DE_OLF and OLF. Given the fact that we use the same information structure and the same random disturbances, the main cause is algorithmic. In particular, OPTCON2 solves nonlinear problems by using an iterative method of linear approximations. This applies for both, the nonlinear system itself and the stochastic information about parameters $\theta$. In contrast, DE addresses the problem without the linear “simplification”, but by using a population of possible realizations of the uncertain parameters directly. In addition, using the least median realization of objective values over a large population of random parameter draws helps DE to mitigate the problem of outliers. All together thid explains the slightly better performance of DE compared to OPTCON2.

To sum up, introducing DE method for solving optimal control problems with passive learning represents a promising research field. The next steps include consideration of active learning strategies and alternative update procedures. Also, more effort should be put to

- increase speed of the DE algorithm, which for the moment being is prohibitively expansive to test larger experiments;
- increase the sample of *K* draws of random disturbances to confirm stability of the results;
- test the framework for more complex problems than the ATOPT model.

## Footnotes

1 See Amman and Kendrick (2003) for an application example.

2 A good example is represented by a model selection problem, where the set of possible combinations $2^k$ quickly explodes in the number of variables *k* [see Savin (2013)].

3 Henceforth we refer to results obtained with OPTCON2 algorithm for open-loop and open-loop feedback simply as OL and OLF.

4 In fact, 1000 generations is a very large number for the particular application in Sect. 4, and the algorithm always converges before reaching that limit.

5 In our application example with $T=5$ this leads to 15 optimization periods. Also instead of finding a solution once for all *T* periods, one has to re-estimate the optimal solution for the updated $\theta ^k$ $T-1$ times.

6 Also, in 56% of cases DE_OLF gives lower values of J than OLF; in 66% of cases DE_OLF gives lower values of J than OL; in 50% of cases DE_OL gives lower values of J than OL.
